# ^18^F-FDG-PET of cardiac sarcoidosis with subcutaneous nodules

**DOI:** 10.1007/s12350-023-03302-7

**Published:** 2023-05-30

**Authors:** Saara Sillanmäki, Saara Iso-Mustajärvi

**Affiliations:** 1 Diagnostic Imaging Center, Kuopio University Hospital, P.O. Box 100, 70029 Kuopio, Finland; 2 University of Eastern Finland, Kuopio, Finland; 3 HeartCenter, Kuopio University Hospital, Kuopio, Finland

## Introduction

Sarcoidosis is an inflammatory condition affecting multiple organs in the body, including the heart. Cardiac inflammation can disrupt normal cardiac function and lead to arrhythmias.

## Case summary

**Figure 1:**
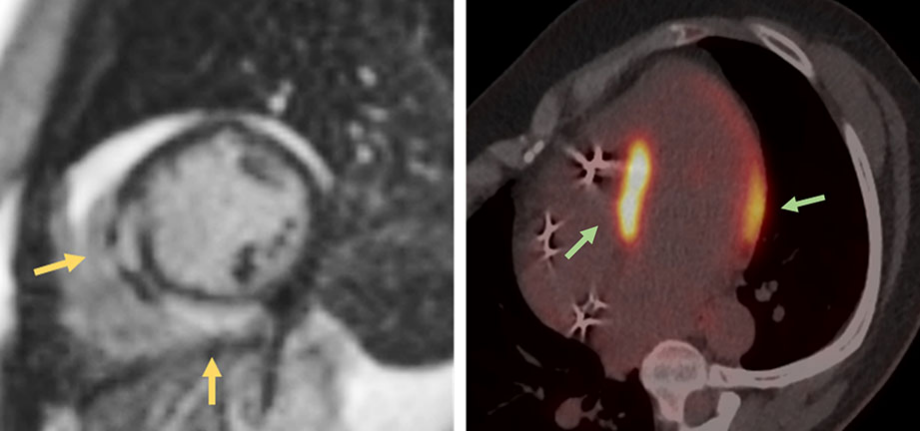
The patient’s initial diagnosis of cardiac sarcoidosis 6 years ago was supported by both cardiovascular magnetic resonance imaging (displayed on the left) and ^18^F-Fluorodeoxyglucose (^18^F-FDG) positron emission tomography scan (displayed on the right). The cardiac MRI shows typical late gadolinium enhancements in the left ventricular septum and posterior wall (indicated by yellow arrows). The ^18^F-FDG PET scan also shows abnormal activity, particularly in the basal septum and lateral wall (indicated by green arrows)

We present a case of a 50-year-old female with no prior medical history who was diagnosed with myocardial sarcoidosis 6 years ago after presenting with symptomatic total block. Magnetic resonance imaging (MRI, Figure 1A) and myocardial biopsy confirmed the diagnosis. The patient was treated with anti-inflammatory medication and a permanent defibrillator was implanted. ^18^F-Fluorodeoxyglucose (^18^F-FDG) positron emission tomography (PET) showed intense activity in the left ventricular walls following a carbohydrate-restricted diet (Figure 1B).

**Figure 2:**
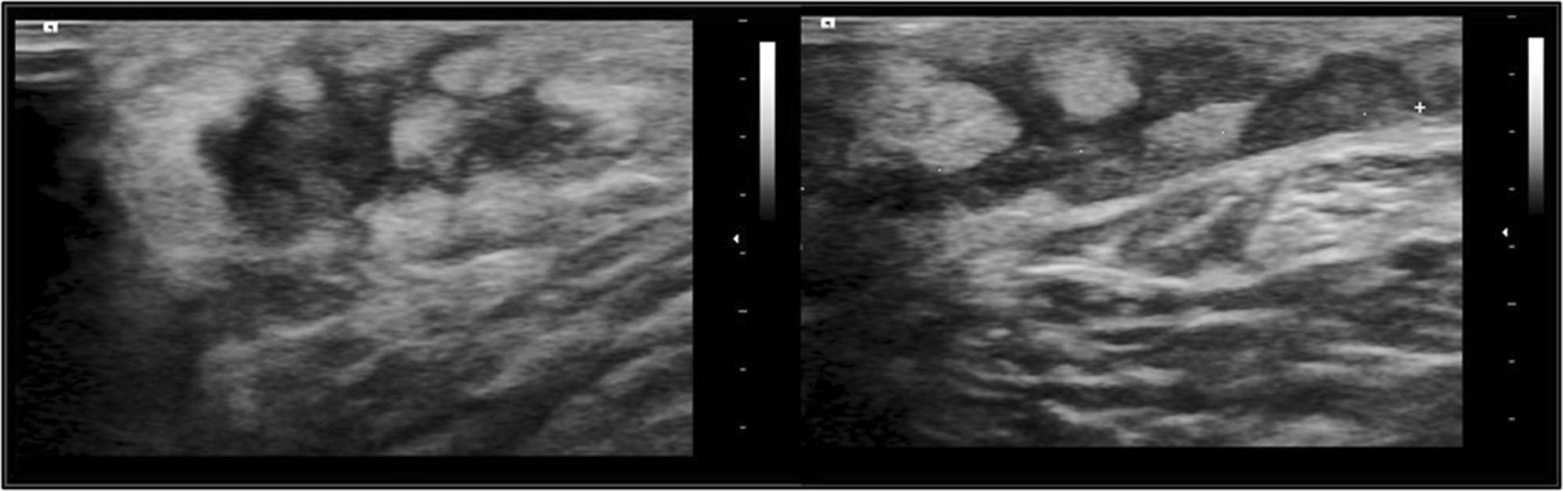
Several subcutaneous oedemic lesions were found in an ultrasound examination autumn 2022. The etiology of the lesions was unsure. Although the cause of these lesions was uncertain, no biopsy was taken at the time. However, a few weeks later, a positron emission tomography scan showed multiple ^18^F-Fluorodeoxyglucose activities in the same areas

The patient was followed for several years, with sarcoidosis medication adjusted according to symptoms, laboratory, and imaging findings. In 2022, she was receiving treatment with infliximab infusion along with prednisolone at a dose of 5 mg/day and methotrexate at a dose of 7.5 mg/week. However, during the spring of that same year, she contracted a COVID-19 infection, and as a result, the infliximab infusion had to be discontinued. No cardiac symptoms were reported, but in late summer 2022, she noticed subcutaneous lumps in her extremities. Ultrasound was performed (Figure 2) and further MRI recommended.

**Figure 3:**
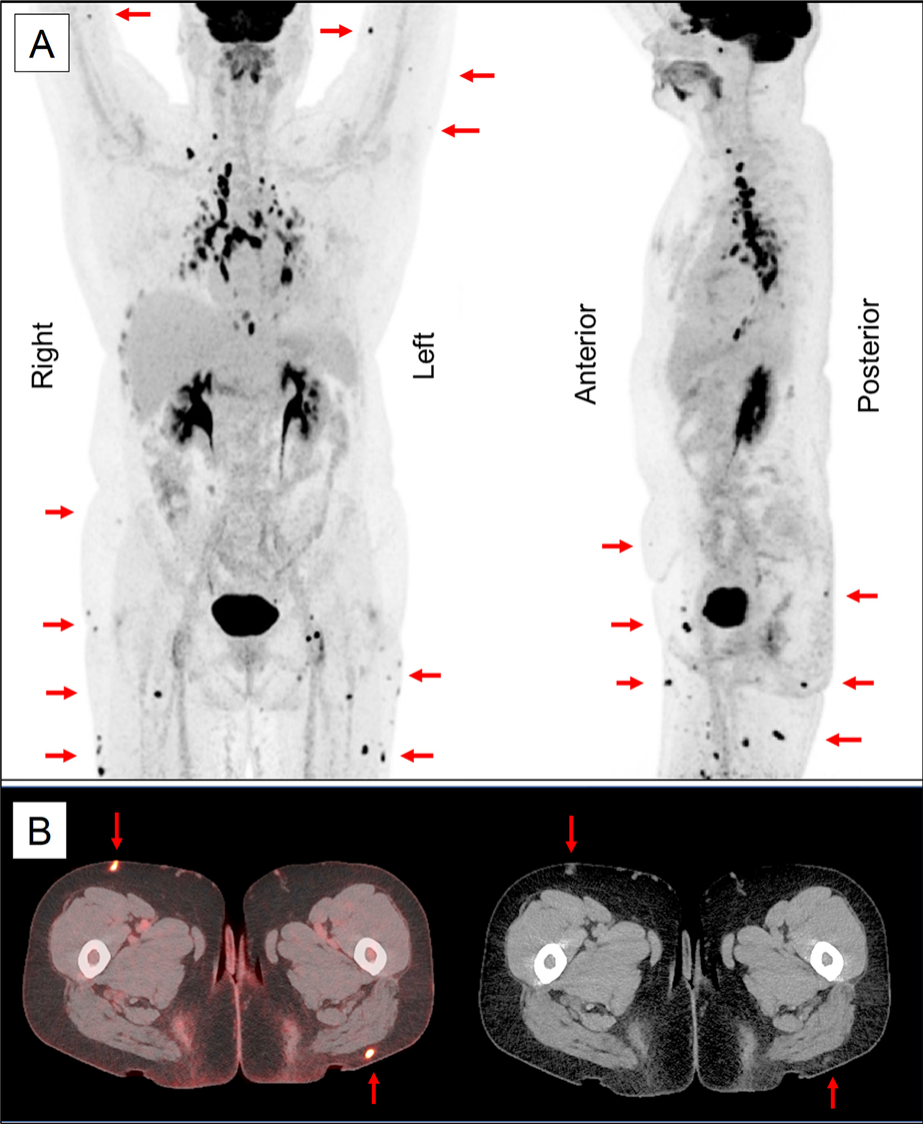
Several abnormal subcutaneous activities (indicated by red arrows) are seen in the latest ^18^F-Fluorodeoxyglucose (^18^F-FDG) positron emission tomography study. Corresponding hyperdense subcutaneous nodules on computer tomography (**B**). Additionally, the patient exhibited lymph node activation in the mediastinum and mild activity in the myocardium. Notably, the ^18^F-FDG activities observed next to the liver were attributed to rib fractures (as seen in **A**)

In autumn 2022, an ^18^F-FDG-PET study prior to her cardiac control showed mild FDG activity in the heart, but active mediastinal lymph nodes (Figure 3A). Multiple ^18^F-FDG uptakes were also detected with corresponding hyperdense subcutaneous nodules on cardiac tomography (Figure 3B). These uptakes were identified as sarcoidosis and were likely the cause of the patient’s subcutaneous lumps. Prednisolone dosing was increased to 20 mg/day and decreased gradually, leading to the disappearance of the subcutaneous lesions on MRI within 3 months.

## Conclusion

The subcutaneous form of sarcoidosis (Darier-Roussy) is a rare entity that should be considered in patients with sarcoidosis and new subcutaneous lumps^1^. It is important to consider this specific entity in patients with sarcoidosis who present with new subcutaneous lumps.
